# 

**Published:** 1853-07

**Authors:** 


### CONTENTS.

ORIGINAL COMMUNICATIONS :— PAGE

ART. I.—The Physiology of Epilepsy and of Paroxysmal Apoplexy, Paralysis, *M*ania, &c. By Marshall Hall, *M*.D., F.R S. 145

ART. II.—Cold Water in Dysentery. By F. Blade, M.D. 152

ART. III.—Pneumothorax with Effusion, Post Mortem, &c. By Ira E Oatman, M D, of Sacramento City, California. 155

ART. IV.—Oxalate of Lime, and its relation to certain forms of Neuralgia. By H A Johnson, M D. 157

An Inquiry, Critical and Experimental, into the Pathology of Fever. By N. S. Davis, M. D. 161

SELECTIONS :—

Editorial Correspondence of the Charleston Medical Journal and Review 177

Relations of Vaccination and Inoculation to Small Pox, 182

Relations of Menstruation, Lactation, and Conception, 185

Dr. Bernard's Experiments on the Elective Elimination of certain Substances by the Secretions. 185

Pathology of Asphyxia. By Thomas Johnson, M. D. 188

On the Influence of Posture in the Treatment of Epilepsy. By Dr Marshall Hall. 196

On the Action of Subnitrate of Bismuth. By Dr. Filippo Lussanna. 197

Letter of Orfila to the Director of the School of Pharmacy of Paris. 199

On Cramp or Spasm. By P. J. Murphy, M. D. 201

Tracheotomy for Opiate Poisoning. 205

Have we a Homœpath among us? 206

EDITORIAL:—

What to observe at the Bedside and after Death in Medical Cases. 207

Principles of Botany. 209

The Virginia *M*edical and Surgical Journal. 210

The Medical Chronicle, or Montreal Monthly Journal of Medicine and Surgery. 212

Respiration subservient to Nutrition. 212

Proceedings of the Medical Association of the State of Alabama. 213

Abstract of the Proceedings of the Cook County Medical Society. By De Laskie Miller, M. D. 217

American Medical Association. 220

Fish of the West. 224

